# Extended Harvest Date Alter Flavonoid Composition and Chromatic Characteristics of Plavac Mali (*Vitis vinifera* L.) Grape Berries

**DOI:** 10.3390/foods9091155

**Published:** 2020-08-21

**Authors:** Ana Mucalo, Edi Maletić, Goran Zdunić

**Affiliations:** 1 Institute for Adriatic Crops and Karst Reclamation, Put Duilova 11, 21000 Split, Croatia; goran.zdunic@krs.hr; 2 Department of Viticulture and Enology, Faculty of Agriculture, University of Zagreb, Svetošimunska cesta 25, 10000 Zagreb, Croatia; emaletic@agr.hr; 3 Centre of Excellence for Biodiversity and Molecular Plant Breeding, Svetošimunska cesta 25, 10000 Zagreb, Croatia

**Keywords:** *Vitis vinifera* cv. Plavac Mali, flavonoids, anthocyanins, tannin, flavonols, harvest date, CIELab color space skin parameters, green berries

## Abstract

This study delivers a comprehensive flavonoid fingerprint profile, physiochemical and external color characterization of Plavac Mali grapes through four harvest dates at two distinct vineyards (Split and Zadar) in the Eastern Adriatic region. The experimental harvest lasted 56 days, at total soluble solids content from 18.4 to 22.4°Brix in Split and 16.8 to 20.4°Brix in Zadar. Patterns of 27 skin and seed flavonoid compounds at each location indicate unique flavonoid composition of berries at each harvest date. Extended harvest increased six compounds in skin with maximum values of main anthocyanin malvidin-3-*O*-glucoside in H3 (4406.6 and 6389.5 mg kg^−1^, Split and Zadar, respectively) followed by a decrease in October. Peak values of seed and skin catechins and galloylated flavan-3-ol subunits are seen in H1 and H2 at Split, while constantly high values are reported in the case of Zadar, with an incoherent pattern of those in skin extracts. Minimal values of epigallocatechin were detected with an extended harvest date at both locations. Berries of extended harvest dates underwent colorimetric improvements, trough decrease in L*, a*, b* and C characteristics and increase in skin color index for red grapes CIRG. The extended harvest date promotes flavonoid composition, and improves the quality of Plavac Mali grape berries.

## 1. Introduction

Continuous physiological and biochemical changes during ripening at the berry level include synthesis of sugars, and flavonoids namely anthocyanins, flavonols, flavan-3-ols, proanthocyanidins [1,2], condensation of compounds of the same or different groups [3,4,5], participation in reactions as intermediates and in transport processes, interconversions of amino acids, catabolism of malic acids [6,7] and polyphenolics as defense units in oxidative stress mediated reactions [8,9]. At the ripening timeline, synchronization of those processes between berries at the cluster level occurs [10], although a certain degree (10%) of heterogeneity lags behind even in extended harvest dates [11]. The enological potential of the berries depends on the quantitative and qualitative polyphenolic profile of the berries in the time of harvest [1,12,13] and their extraction during vinification [3,5,14]. Harvest before berries reached technological maturity, known as cessation of active sugar accumulation, is associated with fresh wines, low in alcohol, high in acids, with a light and vivid color, thin body, bitter taste and astringent sensations [1,13]. Peak in aromatic and flavor complexity, fruity scents and full body, soft tannins wines are associated to phenolic maturity of the grapes [5,11,15]. Extended hang time on the vines in certain vintages gives extraordinary quality [3,13,16]; however, compromised wine quality potential is reduction of natural acidity, increase in pH, reduction of anthocyanins, shift in color toward violet, reduction in polyphenols and color [11,17] and a prevalence of cooked, jammy and waxy types of aromas and flavors of raisins [1]. Selecting the optimal time for grape harvesting is an important decision in grape growing and wine making. Traditionally, the criteria for estimating precise date of harvesting are based on sugar content or sugars/acids ratio, which is not precise enough for phenolic ripeness estimation. In coastal Mediterranean rainfed viticulture areas, an advance in harvest date of up to three weeks [18] is attributed to the acceleration of grape ripening [11,19], through desynchronization of sugar and organic acid metabolism [6] and of sugar and anthocyanin accumulation [20], repression of secondary metabolism at the transcriptomic level and impaired galloylation of proanthocyanidins [6]. High temperatures can inactivate the CO_2_-fixing enzyme RuBiSco, decreasing photosynthesis and the transport rate of sugar into berries and causing a threefold increase in transpiration and stomatal conductance [21]. Blockage of grape ripening is also favored by a severe water deficit [22]. Taken together, those irregularities strongly influence development of varietal flavor potential in the last stages of ripening [23] and can reduce the quality and typicity of existing wine styles of traditional varieties. Extended harvest date exhibit increased concentration of anthocyanins and proanthocyanidins [1,13] but it is also associated with elevated sugar content and low natural acids that consequently increase pH. These grapes are difficult for performing alcoholic and malolactic fermentation. Produced wines are unbalanced with high volatile acidity [11,24], reduced color intensity and increased astringency. Furthermore, high alcohol leads to suppression in aromatic complexity of wines [25].

Flavonoid compounds are important markers of authenticity and typicity of the variety, geographic origin [26], vintage and type of wine [27]. Nowadays, well-structured red wines, rich in tannins with 14 to 16 (% *v*/*v*) alcohol and pH 4 are frequent due to climate change and viticulture techniques that favor production of grapes with high total soluble solids (TSS) content [20]. Several techniques have been proposed for a partial solution that simultaneously increases acidity and decreases pH and alcohol content of wine through a blending approach: early harvest date, sequential harvest regime and production of “green harvest wine” [11,13]. Wines produced from grapes that did not reach phenolic ripeness are less colored and more bitter and astringent, due to reduced anthocyanin and skin proanthocyanidin and greater seed proanthocyanidin extractability [13], which negatively influences the sensory profile of wine [28]. Harvest beyond phenolic ripeness to achieve better coloration and velvety tannins often results in unbalanced wines high in alcohol, low acid content, and high pH, with a higher proportion of seed proanthocyanidin extraction linked to wine matrix composition [11,14]. Such wines are also associated with sluggish fermentation or problematic malolactic fermentation and reduced wine aromatic potential without must N-supplementation [1].

The production of well-structured, balanced wines from the Plavac Mali grape is becoming very challenging in the warm Mediterranean coastal region of Croatia due to climate change. Plavac Mali is a late-ripening red variety used to produce premium red wines on the islands and coastal region of central and south Dalmatia in Croatia. It is characterized by low sensitivity to bunch rot, firm skin and high concentrations of anthocyanins. The seeds and skins of Plavac Mali are rich in flavan-3-ols, catechin, epicatechin and dimers. Recent studies on this variety pointed out that berry proanthocyanidins have high impact on the sensory perception of astringency and bitterness in Plavac Mali wines [29]. Very often, mouthfeel sensory attributes of Plavac Mali wines are described as too harsh or very astringent. Generally, there is decline in seed and skin proanthocyanidins during ripening that complicate finding the appropriate harvest moment for Plavac Mali. A certain degree of heterogeneity in the ripeness of berries in Plavac Mali at the time of harvest has been reported several times [30,31], and green berries can seriously impair the quality of the wine. Unripe green berries are an unexploited source of phenols although those are found as useful as functional ingredient [32] chemical and sensory effect of those on high-quality wines has yet to be clarified.

Although some qualitative responses are known for varieties of a wide range of cultivation further remain unknown for local varieties in their specific terroirs. Given the gaps in knowledge, the aims of this research were to determine the flavonoid profile and chromatic characteristics in Plavac Mali grape berries at four harvest dates, and two vineyards in Eastern Adriatic region of Croatia. Immature (green) berries were separated from first harvest date, and analyzed to determine sugar, acid concentration, pH, potassium, and flavonoid composition.

Understanding flavonoid composition diversity at harvest dates and the answer of varietal grape berry metabolism at different environments are valuable information important for adjustment of agricultural practice and optimization of wine production with enhancing the expression of regional characteristics by managing the flavonoids present.

## 2. Materials and Methods

### 2.1. Chemicals

All chemicals used were of high-performance liquid chromatography (HPLC) analytical grade. Acetonitrile was purchased from from J.T. Baker (Deventer, The Netherlands). Formic acid and orthophosphoric acid (85% *w*/*w*) were purchased from Fluka (Buchs, Switzerland) and ethanol from Kemika (Zagreb, Croatia). Phenolic standard compounds of analytical standard grade used for identification and quantification were: delphinidin-3-*O*-glucoside, cyanidin-3-*O*-glucoside, peonidin-3-*O*-glucoside, malvidin-3-*O*-glucoside, epigallocatechin, procyanidin B1, procyanidin B2, rutin (quercetin 3-*O*-rutinoside) and myricetin from Extrasynthese (Lyon, France); (–)-epicatechin, (+)-catechin and (–)-and epicatechin-gallate from Sigma-Aldrich (St. Louis, MO, USA); kaempferol, quercetin and isorhamnetin from Fluka (Steinheim, Germany) and quercetin-3-*O*-glucoside from Sigma (St. Louis, MO, USA).

### 2.2. Environmental Conditions and Vineyard Design

Experiments on cv. Plavac Mali (*Vitis vinifera* L.) were conducted in two germplasm repository vineyards located in different Dalmatian wine sub-regions: Split–Duilovo (43°30′13.96″ N 16°29′56.467″ E, 14 m asl), located in central Dalmatia, and Zadar–Baštica (44°09′25.6″ N 15°26′12.6″ E, 120 m asl), located in northern Dalmatia. The climate of the experimental area is Mediterranean, with hot and dry summers and rainy winters. The Split vines were grafted onto Boerner (*V. riparia × V. cinerea*) rootstock in 2005 and trained on a bilateral spur-pruned cordon system (eight buds per vine). Vine spacing is 2.0 m (row) and 1.0 m (vine). The Zadar vines were grafted onto Kober 5BB (*V. berlandieri* × *V. riparia*) in 2008 and trained on a unilateral spur-pruned cordon system (eight to 10 buds per vine). Vine spacing is 2.2 m (row) and 1.1 m (vine). Row orientation of both vineyards was north south. The vines of both experimental vineyards were trellised to a vertical shoot position. The soil of both rainfed experimental vineyards is classified as brown soil on limestone.

### 2.3. Harvesting, Grape Sampling and Preparation for Analysis

Grape samples were collected for eight consecutive weeks in 2013. Four different harvest dates were conducted starting at Eichhorn-Lorenz phenological stage (E-L) 35, at which 90% of the berries are at veraison, until (E-L) 39, which coincide with over ripeness [33]. Intervals between harvest dates at each location were 14 days, starting on the 22nd August and finishing 3rd October, and starting on the 30th August until 12th October for Split and Zadar, respectively. At each harvest date, 150 kg of grapes were harvested manually.

Representative vineyard samples at each harvest date were ensured by systematic harvest strategy of one vine per each intermediate space composed of six wines in a vineyard [1]. Immediately after harvest, the grapes were moved to the cooler, their health status was evaluated visually and the whole grape mass was homogenized. A sampling strategy of the smallest possible number of berries from the maximum number of bunches was applied to ensure inter- and intra-homogeneity of the sample at the cluster level. All green berries of 1 harvest date were carefully separated by scissors from the whole grape amount, and were used for physiochemical and flavonoid composition analyses. A total of 1200 randomly selected berries with intact pedicels were sampled each harvest date by taking equal numbers of berries from each cluster. Berries were divided into four groups, each composed of three repetitions per 100 berries. Two separate, randomly selected groups of berries were used for physicochemical and flavonoid analyses. Berry samples for high performance liquid chromatography with diode array detection (HPLC-DAD) identification and quantification of flavonoid compounds were stored at −20 °C for two months.

### 2.4. Analysis of Physicochemical Components of Fresh Juice

Three 100 berries replicate lots of berries per each harvest date were immediately weighted (g) and processed into juice for rapid chemical analysis of basic maturity indicators by manually squeezing the berries without damaging the seeds. Chemical analyses according to the International Organisation of Vine and Wine [34] official methods of analysis were conducted within 24 h of harvest to avoid the negative influence of temperature on the concentration of organic acids. On each of three juice samples, the total soluble solids (°Brix) with a portable density meter (RHW-25/Brix(ATC)), the total acid content by titration to the point of equivalence with 0.1 M NaOH and the bromothymol blue indicator (expressed as g L^-1^ tartaric acid) and pH measured by pH-meter (Metrohm 728, Herisau, Switzerland) were analyzed. Composition and concentration (g L^-1^) of D-glucose and D-fructose in juice was measured using an enzymatic test (K-FRUGL, Megazyme International, WicklowCity, Ireland) according to the recommended spectrophotometric method [34]. Total free amino acids containing the primary amino group were quantified according to an optimized version of the original spectrophotometric method published by the authors of [35] using enzymatic tests (Megazyme K-PANOPA International, Wicklow, Ireland). All spectrophotometric assays were run using a Varian DM 200UV-VIS spectrophotometer and a 1-cm path quartz spectrometer cuvette. Analysis of total potassium concentration was carried out using a flame photometer (Sherwood Scientific Ltd, M410, Cambridge, UK) as recommended [34]. After dilution of the juice sample with deionized water, it was filtered through a cellulose paper filter (Whatman Grade 540, Whatman, Buckinghamshire, UK) and injected directly into a flame photometer. Thermal degradation of the compounds at a flame temperature of 1500 to 2000 °C was achieved with a mixture of propane-butane gas, liberating the potassium atoms, which absorb heat energy and pass into a state of excitation. When the atoms return to their original, lower-energy state, radiation energy is emitted at λ = 766 nm in the violet part of the electromagnetic spectrum. Potassium concentration was calculated from the reading value on the tribal detector, the calibration curve, and multiplication by the dilution factor (FR = 50); the results were expressed in mg potassium L^−1^.

### 2.5. Organic Acids Analysis

Profiles of individual organic acids (tartaric, malic, citric; g L^−1^) were identified and quantified using HPLC with an Agilent 1100 DAD photodiode array detector (Agilent, Palo Alto, CA, USA). After extraction steps for organic acids from juice [36] samples were filtered using a 0.22-µm Millipore filter and directly injected into the HPLC system. The conditions of HPLC analysis were 0.6-mL/min flow and column temperature 65 °C. The stationary phase for separation of organic acids was an organic cation-exchange Aminex HPX-87H column (300 × 7.8 mm id) equipped with a microguard cation H+ cartridge guard column (Bio-Rad Laboratories, Hercules, CA, USA), while a 0.065% aqueous solution of phosphoric acid was used as a mobile phase. Detection of organic acids was carried out at λ = 210 nm. Data analysis was performed using a ChemStation chromatographic data system (Agilent).

### 2.6. Extraction of Flavonoid Compounds from Grape Berries

The second group of frozen berries was composed of three 100-berry replicate lots for each harvest date which were manually detached from the remaining pedicels; grape skin and seeds were separated, air-dried at room temperature for 48 h and ground to a fine powder of particle size <50 μm by mill (Bosch MKM6003, BSH, Nazarje, Slovenia). Solid liquid extraction of the polyphenolic compounds from the lyophilized skin powder was performed according to a modified method from [37] by repeating two extraction cycles with acidified ethanol as follows. In a first cycle, 10 mL of the extraction solvent ethanol:water:formic acid (70:29:1, *v*/*v*/*v*) was added to the lyophilized skin powder (500 mg) and incubated in the dark for 24 h. After maceration, the extraction mixture was centrifuged 15× *g* min at 1814 relative centrifugal force (rcf) (Centric 322A, Tehtnica, Železnik, Slovenia); the resulting supernatant was separated into a falcon PVC tube, and the sediment was extracted once more. In the second cycle, 10 mL “acidified ethanol” (the extraction solvent ethanol:water:formic acid (70:29:1, *v*/*v*/*v*) was added to the sediment and after 30 min incubation in an ultrasonic bath (Sonorex RK 100H, Bandelin, Germany), the extraction mixture was centrifuged 15× *g* min at 1814 rcf. The supernatants from both cycles were combined in a large falcon PVC tube and stored at 4 °C until a concentration step of 12 min on a rotary evaporator (HEI-VAP Advantage, Heidolph Instruments, Schwabach, Germany) at 40 °C under vacuum to remove the ethanol solvent phase. The residue obtained by removing the ethanol was transferred to a 10-mL volumetric flask and supplemented with eluent A (water:phosphoric acid, 99.5:0.5, *v*/*v*).

Solid liquid extraction of flavan-3-ol monomers and dimers from seed powder (125 mg) was performed with 10 mL of extraction solvent acetonitrile:water:formic acid (20:79:1; *v*/*v*/*v*). The extraction mixture was incubated 2 h at 50 °C on a magnetic stirrer (RCT basic, IKA, Staufen, Germany) and 10 min in an ultrasonic bath (Sonorex RK 100H, Bandelin, Germany). The solution was centrifuged 10× *g* min at 3500 rcf (Centric 322A, Tehtnica, Železnik, Slovenia) to precipitate solid particles. The supernatant was transferred carefully to a 10-mL volumetric flask and supplemented with solvent A (water:phosphoric acid, 99.5:0.5, *v*/*v*) to the label. The final volume of 2-mL skin and seed extracts was filtered through a Phenex-PTFE 0.20 μm syringe membrane filter (Phenomenex, Torrance, CA, USA) directly into the HPLC vial.

The individual stock solutions of each standard for calibration curve was prepared by dissolving the flavonoid standard in HPLC-grade methanol and diluting with a previously prepared synthetic wine (hydroalcoholic solution of 12% *v*/*v* ethanol and 3.5 g L^−1^ tartaric acid, pH 3.5 reached by addition of 1 M NaOH) [37]. The calibration curves were made in five points.

### 2.7. Analysis of Flavonoid Compounds by HPLC

Individual flavonoid compounds from grape seed and skin extracts were measured using high-performance liquid chromatograph (HPLC) equipped with two detectors: diode array (DAD), and fluorescence detector (FLD; Agilent 1100, Palo Alto, CA, USA) [37]. Separation of the compounds was performed on a Luna Phenyl-Hexyl column (Phenomenex, Torrance, CA, USA) (250 × 4.6 mm i.d., 5 μm particle size) with a Phenyl guard column (4.0 × 3.0). The column was thermostated at 50 °C and the injection volume for all samples was 20 μL. The gradient solutions used for eluting were two mobile phases: (A) water/phosphoric acid (99.5/0.5, *v*/*v*) and (B) acetonitrile/phosphoric acid/water (50/49.5/0.5, *v*/*v*/*v*). The flow rate was 0.9 mL min^-1^. The linear gradient for eluent B was: 0 min, 0%; 7 min, 20%; 35 min, 40%; 40 min, 40%; 45 min, 80%; 50 min, 100%; 60 min 0%. Analysis of one sample lasted 64 min. Detection of eluent flavonoid compounds was performed on a DAD detector at different wavelengths: flavonols at 360 nm and anthocyanins at 518 nm. Flavan-3-ols were quantitated using a more sensitive fluorescence detector at wavelength excitation λex = 225 nm and wavelength emission λem = 320 nm. Peak identification of individual flavonoid eluate compounds was performed by comparison of retention times, DAD and the fluorescence spectrum with the external standards as described [37,38]. The quantitative values of the identified compounds were calculated using the calibration curves and the peak area of the corresponding standard compounds made by external standard analysis and expressed in mg kg^−1^ dry skin and mg kg^−1^ dry seeds.

### 2.8. Colorimetric Analyses (CIELab)

Berry skin color of previously cotton cloth-cleaned, randomly chosen 100 berries per repetition was evaluated measuring CIELab color space parameters a* (reed/green), b* (yellow/blue), L* (lightness (0, black; 100 white)) using the reflectance spectrophotometer Chroma Meter CR-400 (Konica Minolta, Osaka, Japan) and SpectraMAgic software (NX Lite, ver 2.0). The colorimeter was set to measure reflectance using the llluminant D65 and 2° observation angle. From Cartesian coordinates a* and b*, the cylindrical coordinates h° (hue angle) and C (chroma, relative saturation) are specified. Hue value (h°) represents angle between the hypotenuse and 0° on a* axis of the color wheel that expresses the color tone (+a* axis = 0° (red color); 90° + b* (yellow); 180°-a* (green) and 270°-b* (blue) 0°) and is calculated according formula h° = arctan (b*/a*). Chroma measures intensity or purity of color and is calculated as C = (a*^2^ + b*^2^)^1/2^. Chroma values of represent the distance form grey color toward a pure chromatic color (0, achromatic). The color index for red grapes (CIRG) is calculated as (180 −h)/(L* + C) [39]. All measurements were performed in triplicate, and every berry was measured at the central position at equatorial belt of each berry. The colorimeter was calibrated on standard white calibration plate (X = 0.3183, Y = 86.9, y = 0.3355) before each series of measurement.

### 2.9. Statistical Analysis

A two-way analysis of variance was performed to analyze the significance of the main effects and the interaction between harvest dates and locations. A one-way analyses of variance (ANOVA) and mean separation using Stats-Fisher’s LSD test (different letters account for significant differences at *p* ≤ 0.05) was conducted to detect differences among the four different harvest dates. Analyses of variance were conducted in SAS (SAS Institute, Inc., Cary, NC, USA). The results of all physiochemical and flavonoid parameters are expressed as the average ± standard deviation. All quoted uncertainty is the standard deviation of three replicates of one treatment. Pearson correlation (R) was performed to obtain the degree of correlation between the chromatic CIELab outer color data and individual anthocyanin compounds. The significance of the linear relationship between the two variables is expressed by Pearson’s correlation coefficient in the range of values from −1 to +1. The correlation of each pair of variables was estimated using 24 pieces of original data.

## 3. Results

### 3.1. Climate Conditions

Over the four different harvest dates grapes grown in Split were exposed to higher average monthly temperatures and lower total annual rainfall (Supplement Figure S1). At the beginning of sampling (August), the weather conditions at Split were very hot and extremely dry (6.2 mm of rainfall), mean temperature was 27.6 °C with 5 days in which the temperature exceeded 30 °C, that can stop or promote the degradation of the color matter. On the contrary, the mean temperature at Zadar (August) was 25 °C and total rainfall was 48.9 mm. In September, the average monthly temperature was 21.8 °C and 19.2 °C, at Split and Zadar, respectively. In September, the end of the extremely dry 57- and 51-day period of continuous drought occurred, with a total of 70.3 mm of rainfall in Split, and 102.2 mm in Zadar.

### 3.2. Physicochemical Grape Berry Composition

The analysis of physico-chemical composition of Plavac Mali berries revealed a significant harvest date effect for weight, total soluble solids (TSS), total acidity (TA), pH, potassium, individual organic acids (citrate, tartarate and malate), fructose and glucose–fructose ratio (G:F; Table 1). Concentrations of glucose in berries from Split and total free amino acids containing the primary amino group (PAN) in berries from Zadar were stable regardless of harvest date. Plavac Mali berries reached a peak weight (g) value at the third harvest date at Split and stable values from the second to fourth harvest date at Zadar. Similar tendencies and average increase, variations listed (in parentheses), in TSS (4 and 3.6°Brix), pH (0.37 and 0.23) and decrease in TA (2.8 to 2.5) occurred in berries for Split and Zadar, respectively, over the four harvest dates (six weeks at each location). Split berries reached constant TSS values of 20°Brix at H2 and H3, while those from Zadar, at H3 and H4. Berries from both locations reach constant values of grape juice total acidity (g L^−1^) at the latest harvest dates (H3 and H4). The pH values exceeded 3.5 from the second and subsequent harvest dates at Split, while berries from Zadar reach this critical value only at the fourth harvest date. The concentration of the major cation in grape juice, potassium, varied in a sigmoidal pattern, reaching its greatest values at the second and fourth harvest dates, with a minimum of 1274.3 mg L^−1^ at H3 in Split fruit. A different trend: constant potassium concentration from second harvest onwards occurred at Zadar.

Citric acid was present at a constant concentration with a peak at H4 and malic acid decreased from H1 to H4 at both locations, although there were greater initial concentrations and rates of malic acid consumption in berries from Zadar. The concentration of tartaric acid was constant in Split grapes, except at H3, when the concentration of this primary grape organic acid reached maximal values of 4.3 g L^−1^. In contrast, concentrations of tartaric acid decreased gradually from 5.7 (H1) to 4.4 g L^−1^ (H4) in the Zadar grapes. The glucose and fructose concentrations and glucose–fructose ratio remained relatively constant throughout the harvest dates, with the main changes occurring at H2 at both locations and increased fructose afterwards. The concentration of total free amino acids containing the primary amino group < 128 g L^−1^ remained constant regardless of harvest date and location, with the single exception of H3 berries from Split.

Growing location had a significant impact on the berries’ physico-chemical components, excluding the impact on citric acid and glucose–fructose ratio. Significant two-way interaction effects of harvest date × location factors were observed for physico-chemical components, excluding total acidity (g L^−1^), citric acid (g L^−1^), fructose (g L^−1^) and glucose–fructose ratio.

### 3.3. Evaluation of Grape Berry Flavonoid Composition

Harvest date had a significant impact on the concentrations of individual anthocyanin glucosides in Plavac Mali grape skin extracts (Table 2). Dynamic patterns of 3-*O*-glucosides of delphinidin and malvidin were the same at both locations. The primary anthocyanin, malvidin-3-*O*-glucoside, reached peak values of 4406.6 and 6389.5 mg kg^−1^ at H3 in berries from Split and Zadar, respectively. Petunidin-3-*O*-glucoside reached peak values at H4 and H3 in extracts from Split and Zadar, respectively. Anthocyanins of the cyanidin biosynthetic group peaked at H2 at Split and H4 at Zadar. Skin extracts of berries grown at the two locations differed significantly in their concentrations of anthocyanin glucosides. Higher concentrations of all individual anthocyanins were detected in berries from Zadar, except for anthocyanins of the cyanidin biosynthetic group, which was less abundant at the early harvest dates at this location. The concentration of malvidin-3-*O*-glucoside at H1 in berries from Zadar was similar to that of berries from the latest harvest dates at Split. Anthocyanin profiles from both locations were similar for the delphinidin biosynthetic group, but those for the cyanidin biosynthetic group were different. In this study, only non-acylated forms of anthocyanins glycosides are identified and quantified (Supplement Figure S2); as in a previous study, it was evident that those were dominant due to their low stability over acylated forms highly influenced by pH, light, and temperature.

Harvest date had a significant impact on the flavonol profile of Plavac Mali grape skin extracts (Table 2), except for the hyperoxide concentration, which was stable at Split. Monoglucoside froms of myricetin and isorhamnetin both increased to the final harvest dates. Other flavonol compounds showed different dynamics depending on location. Rutin decreased over time at Split, but increased at Zadar. The highest concentration of rutin in grapes from Zadar (16.6 mg kg^−1^), detected at H3 was three times lower than that in H1 berries from Split (50 mg kg^−1^). Opposite patterns showed a significant decrease and increase of the most abundant flavonol, quercetin-3-*O*-glucoside, decreased under the conditions of Split but increased at Zadar. Concentrations of kaempferol-3-*O*-glucoside had a typical sigmoidal trend in grapes from Split, but increased over time at Zadar. There were significant differences in flavonol concentrations by location in the grape skin extracts, except for isorhamnetin-3-*O*-glucoside. Berry skin extracts from Split had higher flavonol compound concentrations except for the 3-*O*-monoglucosides of quercetin, kaempferol and isorhamnetin, which were more abundant in the berries from the later harvest dates (H3 and H4) at Zadar.

Harvest date significantly affected the flavan-3-ols in Plavac Mali grape skin extracts. The most abundant flavan-3-ol monomer was epigallocatechin and the most abundant dimer was B1, regardless of harvest date or location. The lowest concentrations of epigallocatechin and catechin occurred at H4 at both locations. Gallocatechin increased with delayed harvest date at Split. There were no significant differences in concentrations of gallocatechin, epigallocatechin and catechin in H1 and H3 grape skin extracts from Zadar. Epicatechin followed similar patterns of increase, reaching stable values at later harvest dates at both locations. Different patterns of flavan-3-ol dimers across harvest dates occurred at the two locations. Berry skin extracts at the earliest harvest dates (H1 and H2) from both locations had more dimers, except that B2 and B4 peaked at H3 and H4 at Zadar. Growing location had a significant impact on flavan-3-ol composition of grape beery skin extracts, except for B3.

Harvest date had a significant impact on the flavan-3-ol profile of Plavac Mali seed extracts (Table 3) from grapes grown at Split, except that the concentration of dimer B2 remained constant across harvest dates. In contrast, the concentrations of monomeric and dimeric flavan-3-ols in the Plavac Mali seeds from Zadar were constant after veraison, regardless of harvest date. There were small differences in epigallocatechin and the catechin. The most abundant flavan-3-ol monomers were catechin and epicatechin. Seeds from earlier harvest dates (H2 and H3) had significantly more catechin, epicatechin and epicatechingallate than seeds of the two final harvest dates (H3 and H4). The concentrations of gallocatechin and epigallocatechin were constant during ripening, with a significant decrease in overripe grape seeds (H4). Concentrations of flavan-3-ol dimers were constant after H1, except for the dimers B1 and B4. The concentration of B1 dimer varied during ripening without a clear trend, while B4 decreased at the final harvest dates (H3 and H4). Growing location had a significant impact on the flavan-3-ol composition of the grape beery seed extracts. Seeds from Zadar had higher concentrations of all flavan-3-ols, although these differences were less pronounced with gallocatechin.

There was a significant two-way interaction effect between harvest date and location for all anthocyanin monoglucosides, flavonols and monomeric and dimeric flavan-3-ol compounds in the grape skin extracts, excluding the B1 dimer. No significant two-way interaction effect between harvest date and location was present for monomeric and dimeric flavan-3-ol compounds in grape seed extracts. The effect of factor “harvest date” on those compounds in grape seeds did not vary significantly from one location to the other.

### 3.4. Characterization of Green Berries

Green berries greatly differed from the red ones in H1 harvest date from both vineyards in all quality variables suggesting their immature state of ripeness. The analytical parameters of the green berries juice were: total acidity (18.06 and 18.51 g L^−1^ as tartaric acid), pH 2.87, L-malic acid 7.88 and 10.67 (g L^−1^), sugar content (10.71 and 8.94 °Brix), respectively for Split and Zadar, as seen in Table 4. No significant difference was found in pH, total acidity, potassium, tartaric acid, glucose and fructose content or morphological parameter of the mass of 100 green berry seeds randomly selected from two observed locations. Green berries from Zadar are characterized by higher total weight (150) of berries, average weight of one berry, content of malic acid, PANopa and lower total number of seeds, total weight of seeds, sugar content, citric acid, while succinic acid was not even detected. Two groups of berries differed in polyphenolic profile: berries from Split had a greater concentration of flavonols, flavan-3-ol monomer concentration of catechin and epicatechin and nonflavonoid stilben compound of resveratrol glucoside. No significant differences were observed for gallocatechin, epigallocatechin and dimer B1, B2 and B4 concentration. Notably, a large difference in primary and secondary metabolites in green berries compared to red ones (Table 2) highlight the intense biochemical processes and ripeness heterogeneity of grapes.

### 3.5. Evaluation of External Color: CIELab Skin Parameters

**Figure 1:**
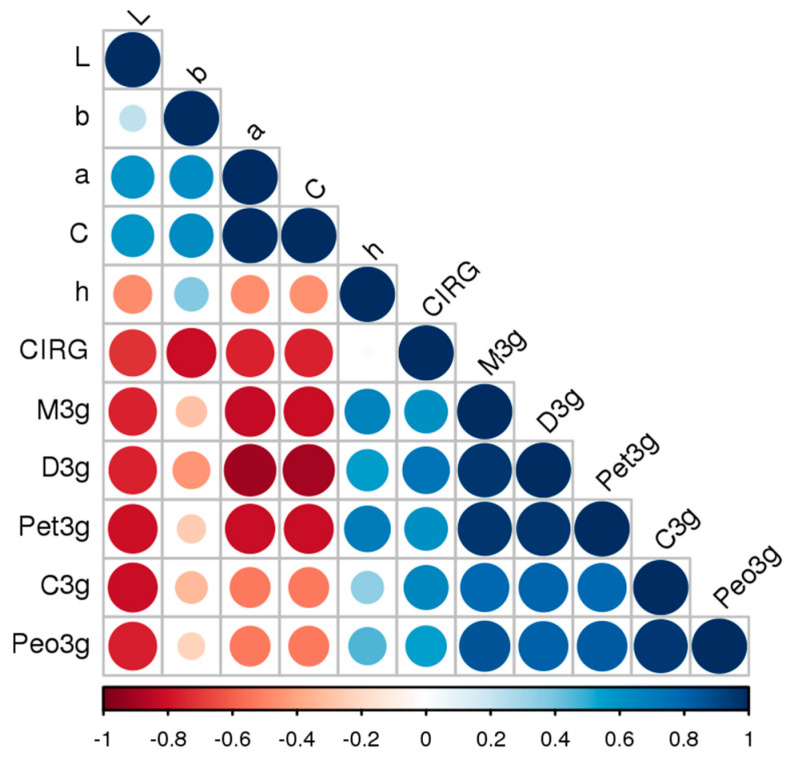
Visualization of Pearson correlation coefficients of CIELab color variables (L*—Lightness, a*—red/green, b*—yellow/blue, C*—chroma, h°—hue angle, CIRG—color index for red grapes), and concentrations of individual anthocyanins (3-*O*-monoglucosides of M3g—malvidin, D3g—delphinidin, C3g—cyanidin, Pet3g—petunidin, Peo3g—peonidin).

Color CIELab quality analysis of Plavac Mali berries from two locations (Table 5) shows significant differences in L*, a*, b*, C, h and CIRG values between four harvest dates and at different vineyard locations. A delay in harvest date leads to a decrease in value of coordinate L*, a*, b* and C. Hue value (6.3 to 10.6) indicate a dark purplish red color of Plavac Mali berries at Split, while values (14 to 17) measured at Zadar corresponds to more vivid red color. Hue (h) follow different patterns with highest values reached at H4 and H1 in the berries from Split and H3 in the berries of Zadar. Taken together these patterns indicate that as ripening progresses the berries become darker in color, with a lower proportion of bright red and higher proportion of blue color, and the decrease in color purity occurs till the late harvest dates (H3 and H4) remaining constant afterwards. On both locations, a significant increase in CIRG value was observed from H1 to H4 with a highest values reached at H4 (6.5 and 6.6 at Split and Zadar). High CIRG values from H1 (5.9) to H4 (6.6) are the indicators of dark violet color of Plavac Mali berries at all stages of sampling of berries regardless of location and with a greater abundance of 3-OH anthocyanins, malvidin-3-*O* glucoside predominance. However, there was no difference in CIRG values of berries from H3 and H4 in the case of Split, and H2 and H3 in the case of Zadar what makes this particular marker individually used as unreliable indicator of the exact timing of harvest. The Pearson correlation coefficients of CIELab outer skin color data and individual anthocyanin-3-*O*-monoglucosides in skin of Plavac Mali are shown in Table 6 and visualization of Pearson correlation coefficients of CIELab color variables is shown in Figure 1. A very strong negative correlation was found between lightness coordinate L* and five main anthocyanin-3-*O*-monoglucosides in the skin from −0.76 for 3-*O*-monoglucosides of delphinidin and malvidin to a −0.81 with cyanidin. A slightly higher negative correlation was between redness cordinate a* and color saturation parameter C with delphinidin based monoglucoside anthocyanins as follows, −0.90 and −0.89 with delphinidin, −0.83 and −0.82 with malvidin and −0.82 and −0.81 with petunidin, respectively. Redness coordinate a* and color saturation parameter C were also significantly correlated with cyanidine based monoglucosides, with correlation coefficient −0.53. There was no significant correlation between yellowness coordinate b* and individual anthocyanin-3-*O*-monoglucosides in skin of Plavac Mali, with the exception of significant correlation with delphinidin-3-*O*-glucoside. Hue angle (h) was a significantly positive correlated to all individual monoglucoside forms except cyanidin-3-*O*-monoglucoside. Correlation analyzes revealed that berry color, expressed as CIRG, was highly positive significant correlated to all individual anthocyanins, with a highest value between CIRG and delphinidin-3-*O*-monoglucoside. There is a highly significant correlation (*p* < 0.0001) of the CIRG with non-methylated, hydroxylated anthocyanins cyanidin- and delphinidin-3-*O*-monoglucoside and significant correlation (*p* < 0.05) with methylated anthocyanins peonidin-, petunidin-, and malvidin-3-*O*-glucosides.

## 4. Discussion

### 4.1. Conventional Indicators of the Grape Ripeness

Extended harvest date had a significant effect on conventional indicators of the technological ripeness, increase in °Brix and pH, and decrease in total acidity [1,11,12]. Compared to other studies on international varieties [11], in the period of 42 days a relatively small increase in TSS values of maximum 4°Brix occurred (Split); moreover, a stable concentration of glucose at the last three harvest dates on both locations, and minor changes in fructose indicate a participation of the same in biochemical processes glycolysis [40] favored by stress conditions during ripening [41]. Taken together, significant decrease in weight, increase in TSS, pH, potassium and citric acid in late harvest dates (from H3 to H4) indicating that the berries entered the phase at which mesocarp cell death occurs connected to dehydration and concentration of compounds as a result of the extended harvest date [42]. However, deviation from standard concentration patterns is seen in the case of total acidity, malic and tartaric acids and PAN. Thermal degradation of the dicarboxylic malic acid is well documented and indicates utilization of malate in respiration [6] in the case of extended harvest dates. Moreover, recently an acceleration effect of high temperatures on utilization of malate [43] in supplementing the anaplerotic capacity of the TCA cycle for amino acid biosynthesis over gluconeogenesis was reported [44]. One of precursors of malic acid in late harvest dates is citric acid [45]. An increase in citric acid and decrease in PAN in later harvest date can be attributed to osmotic and oxidative stress/hypoxia related decreased activity of TCA cycle and acceleration of glycolysis [46,47]. A significant decrease in the tartaric acid concentration from H3 to H4 at Split is consistent with the fastest formation of potassium bitartarate at pH 3.7 [36], and refers to a disorganization and cell death in late ripening stages [47]. The positive slope of a double sigmoidal pattern of potassium at H2 is associated with a phloem activity, while at H4 probably to leakage of potassium into the extracellular space [42]. In addition to the potassium affinity of tartaric acid, high concentrations of this cation in Split berries could disrupt the normal transport of malic acid (0.3 g L^−1^ in berries at H4) from the vacuoles into cytoplasm, where normal respiration occurs. The maximum must pH coincided with the maximum potassium and sugar accumulation and with the minimum total acidity in grape berries at both locations. The concentration of total free amino acids containing the primary amino group was stable regardless of harvest date at both locations, except that at H3 at Split.

The primary metabolic set for both locations represented 16% of the metabolites differentially expressed with location, 33% with interaction (location and harvest date), as follows: citric acid, fructose, glucose fructose ratio and total acidity; these factors were not influenced by location but with the development of berries and sugar content at certain harvest date. That observed differences occurred after metabolic shift when berries reached phenol ripeness at H3 in the case of Split, and no clear evidence that berries from Zadar entered this phase within the four harvest dates, confirms that the same genotype varies in terms of the two locations. The results from this experiment are consistent with a hypothesis that abundance and patterns of metabolites during grape ripening were sensitive to environment, climate and soil characteristics of the location [48,49] except for total acidity, citric, malic acid, fructose, and glucose/fructose ratio, patterns of which were the same across vineyards.

There was an accelerating ripening trend [19] with a rapid increase in TSS until stable values were reached at H2 at Split and at H3 at Zadar. Higher temperatures and drier conditions at Split in southern Dalmatia decreased total acidity and increased concentrations of the osmoprotective compounds TSS, glucose, fructose, potassium, consequently increasing pH [23]. Berries from Zadar had higher concentrations of totalacidity, tartaric and malic acid, and lower pH regardless of harvest time than berries from Split, in agreement with reported increased total acidity with greater water availability [50] and humid conditions [23] in northern Dalmatia. While a higher consumption rate of malic acid in berries from Zadar can be attributed to a promotion reduction effect of diurnal temperature differences.

### 4.2. Flavonoid Indicators of Grape Ripeness

#### 4.2.1. Flavonoid Compositional Changes in Grape Skin

Extended harvest date had a significant effect on flavonoid indicators of the phenol ripeness, increase in anthocyanins and decrease in flavan-3-ol monomers of the grape skin and seeds [12]. Although patterns of five anthocyanin monoglucosides increased from H1 to a H4, a peak in main component in Plavac Mali [29,51] malvidin-3-*O*-glucoside was reached at H3 on both locations with a significant decrease afterwards. On the contrary [16], an increase in final products of anthocyanin pathway, malvidin- and peonidin-3-*O*-glucosides, and a decrease in other monoglucoside forms in Cabernet Sauvignon was attributed to an extended harvest date and partly to a late season fungal infections. However, a mismatch in reaching the pattern peak in those compounds due to location is seen in Table 2. The main reason for a decrease in the otherwise thermally stable 3′,5′-dimethylated anthocyanin malvidin-3-*O*-glucoside with extended harvest (H4) could be overexpression of the anthocyanin acyl transferase Vv3AT gene [4] and a greater activity of post-synthesis aliphatic and aromatic acyltransferase enzymes in response to berry temperature stress [52]. This is consistent with preliminary reports of increased malvidin-3-(6″-***O***-coumaryl)-glucoside during ripening in Plavac Mali berries [51]. Significant changes between H3 and H4 in methoxylated anthocyanin forms a decrease of malvidin-3-*O*-glucoside, constant or an increased concentration of others (petunidin- and peonidin-3-*O*-glucosides) and parallel increase in the methoxylation 3′OH quercetin-derivative isorhamnetin-3-*O*-glucoside and a decrease in rutinose 3′OH quercetin-derivative rutin is seen at both locations. Anthocyanin methoxylation during ripening represents a strategy of the grapevine to cope with an abiotic stresses as water availability [53] and temperature [52], while a decrease in flavonol hydroxylation and increase in methoxylation level can be modified by an ambient solar UV radiation level [54]. A negative pattern of anthocyanins in late harvest date [12,55] can be associated to a biosynthesis slow down, and susceptibility to a chemical and enzymatic degradation of anthocyanins with an ortho-di-phenolic group (cyanidin, delphinidin, petunidin-3-*O*-glucoside) due to oxidative stress at the berry level [9,52]. Conversion of free anthocyanins into derived pigments in the skin is another important aspect that should be taken in account [56]. It is important to point out that this analysis is limited in two ways. The first is the limited number of flavonoid compounds examined, yet these were chosen because previous studies demonstrated they were the most highly represented in berries of Plavac Mali [51]. The second is the heterogeneity of berries that was not considered as in [13,30], as all parameters were measured on set of 100 berries in three repetitions, randomly collected and the average value of each parameter is reflected. Different accumulation patterns of flavonols during ripening due to location are seen, dynamic in the case of Split and linear progressive in Zadar. A decrease in flavonol content with an extended harvest date in the case of rutin on both locations, and decrease of quercetin-3-*O*-glucoside and kaempferol-3-*O*-glucoside in the case of Split can be attributed to berry tissue senescence favored by environmental conditions of higher solar radiation of drought-induced bunch zone leaf drying [22]. Kaempferol is proposed as a metabolic indicator of the accumulated solar radiation received by the berry through the progress of ripening and increasing water deficit [57]. Double sigmoidal pattern in the case of Split with the highest kaempferol-3-*O*-glucoside values at H3 and decrease afterwards make it unreliable indicator in our study; moreover, it is not possible with the experimental design of this study to determine impact of each environmental factor that contributed to a difference in metabolite patterns between those two sites. Also, we cannot exclude the possible occurrence of copigmentation reactions in in vivo systems responsible for pigment stabilization and their protective role in preventing the photobleaching of anthocyanins [58,59]. The extended harvest date leads to a decrease in flavan-3-ol monomer content in grape skin. Regarding the different pattern dynamic of catechins, a decrease in predominantly represented epigallocatechin and catechin during ripening with a stable concentration at later harvest in the case of Split, as previously reported in early September [60], and an increase in gallocatechin and epicatechin are mainly attributed to a grape-ripening process [22,61]. On the contrary, there were no significant changes in composition of those four flavan-3-ol monomers in the skin of Sangiovese berries in five points after full veraison, but an analysis of extractable monomers revealed significant changes, showing a decrease in predominantly represented catechin and epicatechin during ripening, counterbalanced by an increase in epigallocatechin and epicatechin gallate [15]. A decrease of flavan-3-ol monomers partly could be attributed to an increase in mean degree polymerization values in grape skin during ripening [56], although an important parameter of intactness within a subcellular compartment must be satisfied [3]. Previous proanthocyanidin structural characteristics indicate a higher size of polymers, lower galloylation and prodelphinidin percentage in Plavac Mali grape skin extracts in comparison to Cabernet Sauvignon and Merlot [29,62]. A decrease in skin proanthocyanidin dimer content with an extension in harvest date on both locations, with the lowest concentrations of each reached in H4, beside the dimer B2 at H1, suggests that it is unlikely that a direct polymerization occurred. Moreover, as it is well known that in an enzymatic oxidation process mediated by polyphenol oxidases, oligomers composed by oxidized flavan-3-ols with specific configurations are formed [8]. Decreased or stable values from H3 to H4 on both locations are consistent with a reduced extractability of those compounds from skin due to negative impact of increased skin cell wall porosity on proanthocyanidin sequestration, and a higher encapsulation and adsorption in the last phases of ripening [3]. The greatest ratio of beneficial prodelphinidin to procyanidin monomer units [29] was detected at H3 or H2 at Split or Zadar, respectively. A high concentration of epigallocatechin, the predominant flavan-3-ol monomer in Plavac Mali grape skin extracts, is followed by catechin and its enantiomer epicatechin, regardless of location or harvest date. There is twice as much prodelphinidin (GC, EGC; with a trihidroxylated–3′4′5′ B ring) as procyanidin (C, EGC; with a 3′4′ dihidroxylated B ring) monomer units regardless of harvest date, suggesting a high-quality potential of Plavac Mali, in accordance with previous research on Cabernet Sauvignon [61]. The amount of prodelphinidin monomer units is an indicator of high-quality potential of variety [13] and high-terroir potential for production of premium red wines favored by climate conditions that provide complete maturity [63]. Different flavan-3-ol monomer and proanthocyanidin dimer composition and quantity reflect location differences, mainly differences in environmental factors. As previously described, the positive impact of sunlight exposure on flavan-3-ol monomer synthetic enzyme genes [64] and water stress status on polymerization degree without impact on galloylation [65,66] and the absence of effect of high temperature on proanthocyanidin accumulation with a moderate effect on galloylation and proanthocyanidin composition [43]. However, in our case, similar values of each flavan-3-ol monomer and dimer compound in the grape skin extracts of two locations are indicators of a stronger ripening-related effect than the environment.

#### 4.2.2. Flavonoid Compositional Changes in Grape Seeds

The predominance of catechin in Plavac Mali grape seeds at early harvest dates at Split and the predominance of epicatechin at later harvest dates [67,68] are consistent with reports of differential rates of decline of individual flavanol monomers after veraison [12]. The maximal monomer and dimer values reached in H2 beside in H1 in case of Split are unusual, but a similar phenomenon was previously reported on Touriga Francesa and could be attributed to rapid oligomerization, that would deplete the monomers [68], as it is quite unlikely to attribute this as a consequence of decrease of total berry or seed weight. Extended harvest dates in the case of Split were characterized by a decrease in flavan-3-ol monomers and procyanidins with the lowest values reached in H4. Stable values of flavan-3-ol monomers and dimers reached in H3 and H4 are similar to previously published research on international varieties [67,69]. The exceptions are gallocatechin and epigallocatechin as values of those are stable whole period from H1 to H3 with a significant decrease afterwards. Negative patterns of those compounds except for biosynthesis slowing down are associated to shift of the balance in the direction of their oxidation and polymerization as previously reported in the case of Cabernet Sauvignon [67,69]. Products of oxidative cross linking are less extractable due to their binding protein and polysaccharide capacity at cellular level [70].

High values and absence of the effect of harvest date on seed flavan-3-ol monomers and dimers are associated with disruption of ripening in the case of Zadar and it is marker of immaturity. The predominance of catechin, epicatechin and procyanidins (B1, B2 and B4), regardless of harvest date, at Zadar can be attributed to a reduced ripeness of seeds mainly due to negative influence of humid conditions [65] on seed desiccation, as previously seen in the case of irrigated berries [66]. Recently [43], a decrease in total seed tannins and changes in other seed compositional parameters was associated with disruption of berry and seed development upon day heating, as heatwaves leads to a disruption in seed development, development of smaller berries and seeds with less tannins. Beside this, lower values of proanthocyanidins in Split can be attributed to a suppression drought effect on available carbon for carbon-based secondary compound biosynthesis in stressed vines [66].

Compositional analysis and quantification of conventional and flavonoid metabolites at four harvest dates from two vineyards found high variability in the various classes of phenolics. Different and unpredictable patterns of phenolic compounds in berries at different harvest dates indicate a possibility to produce a variety of wine types. Higher flavonoid concentrations, a lower sugar content and a higher proportion of total acids with optimal pH values responsible for the production of highly balanced wines are seen at different harvest dates in new Plavac Mali production region of Zadar. However, authentic, premium-quality varietal wine with a strong expression of typical terroir can be only obtained from berries that reach phenolic ripeness, thus those from Split. Despite a good flavonoid potential, wines made from Plavac Mali grapes subjected to prolonged ripening would lack a primary varietal identifier. Variation in the qualitative ratio of flavonoids with the predominance of seed flavan-3-ol monomers and dimers and unclear achievement of phenolic maturity in the berries from Zadar indicate a low quality potential for moving viticulture zones of Plavac Mali production to higher latitudes as a climate-adaptable viticulture strategy. The inability of the late-ripening variety Plavac Mali to reach phenolic maturity, as previously reported for Carignan [71,72], indicates the low phenotype plasticity for this variety and a unique effect of vineyard location and climate conditions on the typicity of its wines. A proposed climate change adaptive strategy of spreading the cultivation of Plavac Mali to new regions would be highly demanding due to their low plasticity and the inability of its berries to reach phenolic ripeness in some cooler locations.

### 4.3. Green Berries

Lower sugar and higher acidity content of green berries could make them useful in maintaining the ethanol content and optimal pH of the future wine [13], but their flavonoid composition and predominance of seed flavan-3-ol monomers and dimers should be taken into consideration. Interestingly, there was a lower concentration of flavan-3-ol monomers and dimers in green berry seeds and skins in comparison to colored berries. Higher amounts of epicatechin and dimer B2 were detected only in the seeds of green berries from Split, while gallocatechin, epigallocatechin and catechin were higher in skins of green berries of both locations that can be attributed to higher proportions of (−-epigallocatechin and (+)-catechin and less polymerised proanthocyanidins in the case of low-density grapes [13].

Generally, these minor differences in flavan-3-ol monomers and dimer concentration and composition of green versus colored berries of early harvest dates make these berries interesting source for green harvest vinification approach [11]. Moreover, this is favored by a minimal impact of green harvest wine (GHW) on wine polyphenols that is function of lower wine pH [13]; non-beneficial is a significant decrease in SO_2_-resistant pigments, especially in the case of greater amount of 43% juice substituted with GHW [11]; however, the inability to sensorially distinguish those wines from regular ones [13], and the fact that differences in SO_2_-resistant pigment fade away after 18 months of bottling age [11], makes them interesting in optimization of acid part must matrix wine before fermentation. Furthermore, this is supported with a higher amount of non-extractable tannins (25%) in under-ripe versus ripe (18%) seeds and lower amount of tannin extracted from under ripe (72%) versus ripe seeds (77%) [14]. A lower concentration of available polyphenols, particularly anthocyanins, flavonols and flavon-3-ol monomers and a lack of reduction factors Nicotinamide adenine dinucleotide(NADH) or Nicotinamide adenine dinucleotide phosphate (NADPH) caused by a strong amino acid metabolism, is a potential problem of GHW and early harvest dates that leads to production of wines high in acetaldehydes [25]. A higher content of rutin and quercetin-3-*O*-glucoside in green berries in comparison to colored berries and higher content in berries from Split than that from Zadar indicate a higher exposure of clusters to solar radiation [57] as there were certain differences in canopy architecture between those two locations that are a confronted with a drought and higher temperatures consequences in the case of Split.

Great differences in the morphology of green berries on the two studied locations indicate a higher seed-to-berry ratio in the case of those from Split (as they had lower berry mass, larger number and weight of seeds), which can be one reason of lagging those berries [10], as berries with a higher seed content enter the ripening phase 4–14 days after those with lower seed content. Moreover, kinetic ripening is associated with a seed-to-berry ratio, and a low ratio led to rapid sugar accumulation and discoloration stage. In Plavac, Mali-cluster green berries were found in first harvest dates on both locations and there were no lagging berries afterwards that is in accordance to altered transcriptional program and enhanced ripening rate of the berries that were lagging in the time of veraison that helped the natural asynchrony in grape berry ripening of Plavac Mali to be overcome through the period to full maturity. The flexibility of ripening program speed through faster completion of transcription program, shifts in gene expression and maturation dynamic of related hormones, increases the rate of progression of physiological maturation of berries that were lagging but transcriptional distances at a certain level exist throughout the ripening period, regardless of the berry class [10]. Berry synchronization is associated with an acceleration of sugar import and metabolism in berries that were lagging, as a consequence of the sugar and hormonal regulation of gene expression.

### 4.4. External Color CIELab Skin Parameters as Indicators of Ripeness

Color CIELab quality patterns indicate that as ripening progresses the berries become darker in color, with a lower proportion of bright red and higher proportion of blue color, color purity decrease till the late harvest dates (H3 and H4) remaining constant afterwards [73,74]. The dark color of berries is associated with a higher content of anthocyanins in the skin in the late harvest dates. A highly significant linear correlation (r = 0.8475 and r = 0.8322, *p* < 0.001) during two seasons have been established between CIRG and total anthocyanin (mg per g) accumulation [75]. Significant increase in CIRG value observed till H4 is in agreement with a previous study of Plavac Mali [51]. High CIRG values are indicators of dark violet color of Plavac Mali berries at all stages of sampling of berries regardless of location and with a greater abundance of 3-OH anthocyanins, malvidin-3-*O*-glucoside predominance [76]. The CIRG exhibits strong linearity with the visual color of berries and is useful tool for distinguishing between sample groups of different external color [39]. However, no difference detected in CIRG values of berries from H3 and H4 in the case of Split, and H2 and H3 in the case of Zadar makes this particular marker, used individually, as an unreliable indicator of the exact timing of harvest.

The evolution of the external skin color indicators is cultivar dependent, and hormone regulation and ecophysiological modulation during ripening plays important role. Recently, the significant influence of S-ABA [75] and 24-epibrassinolide treatment [77] in table grapes on the intensity and development of color is reported. A decrease in L*, C, b*, a* and increase in CIRG values of berries on both locations with extended harvest date suggest an increase in darker violet, slightly less pure color and development form a vivid to a more dull color, through an increase in total anthocyanin content. Lower C* value indicates low color purity and is undetectable to the human eye, but changes in h° value is easily perceived by the human eye as increase is associated with a diversion towards red color [75] and decrease with a development of a more red–violet color of the berry skin [74]. The low hue values, lower than 18, in this experiment indicate a dark color of Plavac Mali berries [74] and different patterns seen between two locations are probably due to a difference in weather conditions during ripening between summer and offseason.

Patterns and magnitude of differences in changes of CIELab parameters were higher in the case of Split than in case of Zadar for all parameters except L* suggesting an acceleration in pigmentation process [75,77] of berries from Split. However, neither one of CIELab parameters is a real indicator of phenolic maturity in our case, as there was no significant differences of each component measured in H3 from those measured in H2 or H4, except for b* in the case of Split and L* and C in the case of Zadar. The lower values of the CIELab coordinate b* in berries of H2 to H4 harvest dates and h and CIRG values at each harvest date from Split. Although modification of CIELab coordinate b* has been associated with differences in concentration of tri-hydroxylated malvidin-, petunidin-, and delphinidin-3-*O*-glucosides and to an increase in of purple/blue pigmentation due to a water stress during ripening [53], we are not able to confirm this with design of our experiment. The deviation from expected is probably result of synergistic effect of the drought and high temperatures in the case of Split as it is a well-known an inhibition effect of high summertime temperatures on biosynthesis [78] and parallel promotion effect on degradation of anthocyanins [9]. The relationship between external CIELab color parameters and individual monoglucosides of anthocyanins at both locations was defined as a highly correlated. Established highly significant (*p* < 0.001) relationships indicated that changes of external CIELab color parameters in skins occurred simultaneously with developmental changes in anthocyanin profile of the skin. The index of ripeness CIRG showed a significant correlation to different groups of hydroxylated and methylated anthocyanins [77]. The practical value of these results is that the changes in skin color can be easily used till one point just for a rough selection between unripe and grape that reached phenolic ripeness; afterwards, changes in CIRG value fade away. Moreover, for a practical implication of this index, a “sense of place” of vineyard should be taken in consideration.

## 5. Conclusions

In this study, we show large differences in qualitative and quantitative flavonoid composition of Plavac Mali grapes as an effect of extended harvest date at two different agro-ecological locations. The extended harvest date increases the total soluble solids, pH, and changes the flavonoid composition in favor of anthocyanins and flavonols. Total acidity and flavan-3-ol monomer and dimer compounds related to a grape skin and seed extracts decreased with the extension of harvest date. Changes in chromatic characteristics occurred simultaneously with developmental changes in anthocyanin profile, but from a practical point of view those parameters can be easily used as indicators just for a rough selection between unripe and grapes that reached phenolic ripeness; afterwards, changes in CIRG value fade away. Moreover, for a practical implication of this index, a “sense of place” of vineyard should be taken in consideration.

Immature (green) berries separated from bunches of first harvest date had a lower sugar and pH, and higher acidity content, however in their flavonoid composition there was a predominance of seed flavan-3-ol monomers and dimers. Interestingly, in comparison to seeds of colored berries of the first harvest date, those were lower in concentration of flavan-3-ol monomers and dimers, except for epicatechin and dimer B2 in the case of Split.

Much of the conventional and flavonoid composition patterns differed between the two locations and could be associated with multiple differences in environmental conditions that may have affected the berries in the two locations, also corresponding interaction with harvest date suggest a need of adjustment of optimum ripeness indicators under the environmental conditions of each individual location. Finding the optimum moment for grape harvest is a technological challenge, as conventional indicators of ripeness (sugar, total acidity and pH) and the polyphenolic indicators (anthocyanins, flavonols and monomer and dimer proanthocyanidin units) split up significantly, with different optimal harvest dates. Conventional indicators were optimal at H2 and flavonoid indicators at H3.

## Figures and Tables

**Table 1:** Effect of the harvest date and growing locations on technological maturity indicators—composition analysis of cv. Plavac Mali grape berries.

	Split				Zadar					
Compound	H1	H2	H3	H4	H1	H2	H3	H4	Location	HD × L
Weight ^1^	221.78 ± 8.40 ^b^	165.48 ± 7.04 ^d^	244.41 ± 6.17 ^a^	185.29 ± 3.53 ^c^	271.09 ± 7.49 ^b^	295.58 ± 1.94 ^a^	297.65 ± 14.82 ^a^	302.16 ± 2.28 ^a^	***	***
TSS ^2^ (°Brix)	18.39 ± 0.40 ^c^	20.68 ± 0.59 ^b^	20.53 ± 0.13 ^b^	22.42 ± 0.06 ^a^	16.83 ± 0.38 ^c^	18.69 ± 0.43 ^b^	20.04 ± 0.17 ^a^	20.38 ± 0.13 ^a^	***	**
TA ^3^ (gL^−1^)	6.76 ± 0.31 ^a^	4.84 ± 0.02 ^b^	4.03 ± 0.09 ^c^	3.98 ± 0.14 ^c^	7.28 ± 0.17 ^a^	5.45 ± 0.07 ^b^	4.73 ± 0.18 ^c^	4.82 ± 0.17 ^c^	***	ns
pH	3.38 ± 0.02 ^d^	3.57 ± 0.01 ^c^	3.63 ± 0.02 ^b^	3.75 ± 0.01 ^a^	3.27 ± 0.02 ^c^	3.42 ± 0.02 ^b^	3.49 ± 0.01 ^a^	3.50 ± 0.02 ^a^	***	***
Potassium (g L^−1^)	1316.36 ± 50.5 ^b^	1784.38 ± 46.3 ^a^	1274.31 ± 28.9 ^b^	1731.28 ± 96.2 ^a^	739.15 ± 13.6 ^b^	1108.49 ± 14.2^a^	1191.04 ± 43.1 ^a^	1174.66 ± 79.9 ^a^	***	***
Citric a ^4^ (g L^−1^)	0.16 ± 0.01 ^b^	0.15 ± 0.01 ^b^	0.16 ± 0 ^b^	0.35 ± 0.07 ^a^	0.14 ± 0.01 ^b^	0.15 ± 0.01 ^b^	0.14 ± 0.02 ^b^	0.34 ± 0.01 ^a^	ns	ns
Tartaric a ^5^ (g L^−1^)	3.49 ± 0.25 ^b^	3.57 ± 0.09 ^b^	4.32 ± 0.03 ^a^	3.88 ± 0.33 ^b^	5.73 ± 0.06 ^a^	5.14 ± 0.08 ^b^	4.88 ± 0.08 ^c^	4.37 ± 0.14 ^d^	***	***
Malic a ^6^ (g L^−1^)	2.17 ± 0.33 ^a^	1.12 ± 0.04 ^b^	0.68 ± 0.02 ^c^	0.25 ± 0.03 ^d^	3.43 ± 0.06 ^a^	2.20 ± 0.05 ^b^	1.58 ± 0.08 ^c^	0 ^d^	***	***
Glucose (g L^−1^)	105.99 ± 7.59 ^a^	118.74 ± 19.81 ^a^	99.36 ± 8.19 ^a^	108.20 ± 7.48 ^a^	82.51 ±7.40 ^b^	98.51 ± 7.07 ^a^	102.49 ± 5.25 ^a^	111.73 ± 8.35 ^a^	*	*
Fructose (g L^−1^)	89.53 ± 21.93 ^a^	58.77 ± 17.50 ^b^	92.28 ± 11.24 ^a^	105.83 ± 11.44 ^a^	68.77 ± 8.74 ^ab^	33.89 ± 12.92 ^c^	52.53 ± 29.13 ^bc^	94.05 ± 3.53 ^a^	**	ns
G:F ^7^	1.21 ± 0.19 ^b^	2.14 ± 0.63 ^a^	1.08 ± 0.05 ^b^	1.03 ± 0.14 ^b^	1.22 ± 0.24 ^b^	3.37 ± 1.81 ^a^	2.34 ± 1.06 ^ab^	1.19 ± 0.08 ^b^	ns	ns
Panopa ^8^ (g L^−1^)	52.61 ± 5.29 ^b^	48.54 ± 6.66 ^b^	75.65 ± 12.55 ^a^	53.23 ± 8.04 ^b^	120.58 ± 21.21 ^a^	127.96 ± 4.08 ^a^	111.44 ± 14.32 ^a^	118.37 ± 11.71 ^a^	***	*

Two-way ANOVA showing mean separation of the conventional components of Plavac Mali grape berries harvested at four dates (H1, H2, H3 and H4) at two locations (L; Split and Zadar) and the interactive effect (I = HD × L) of these factors (ns: not significant; * *p* ≤ 0.05; ** *p* ≤ 0.01; *** *p* ≤ 0.001). Mean values ± standard deviation (*n* = 3) within the same line followed by different letters indicate significant differences according to Fisher’s LSD test at *p* ≤ 0.05 between different harvest dates, separately for each growing location, as determined by one-way ANOVA. ^1^ Weight—total weight/100 berries; ^2^ TSS—total soluble solids; ^3^ TA—total acidity expressed as (g L^−1^) tartaric acid equivalents; ^4^ Citric a:—citric acid; ^5^ Tartaric a—tartaric acid; ^6^ Malic a—malic acid; ^7^ G:F—glucose to fructose ratio; ^8^ Panopa—primary amino nitrogen.

**Table 2:** Flavonoid composition (mg kg^−1^ dry weight) of Plavac Mali grape skin at four different harvest dates at two locations.

		Split				Zadar					
	HD ^1^	H1	H2	H3	H4	H1	H2	H3	H4	L ^2^	I ^3^
Anthocyanins	D3g ^4^	382.9 ± 15.6 ^d^	692.9 ± 12.6 ^c^	812.0 ±15.8 ^b^	855.3 ± 21.2 ^a^	729.2 ± 7.8 ^d^	806.4 ± 6.1 ^c^	1087.7 ± 6.0 ^b^	1126.1 ± 14.6 ^a^	***	***
	C3g ^5^	27.8 ± 1.2 ^b^	41.3 ± 3.9 ^a^	37.6 ± 2.5 ^a^	40.1 ± 3.8 ^a^	24.4 ± 2.4 ^d^	34.1 ± 2.4 ^c^	54.8 ± 4.5 ^b^	68.5 ± 7.7 ^a^	***	***
	Pet3g ^6^	450.3 ± 6.3 ^c^	673.4 ± 16.4 ^b^	666.9 ±10.7 ^b^	853.4 ± 10.0 ^a^	740.1 ± 12.2 ^c^	800.1 ± 4.9 ^b^	1111.0 ± 53.3 ^a^	1081.3 ± 19.8 ^a^	***	***
	Peo3g ^7^	110.7 ± 3.4 ^c^	147.3 ± 3.8 ^a^	132.3 ± 3.4 ^b^	127.0 ± 3.7 ^b^	103.3 ± 6.2 ^d^	133.9 ± 2.7 ^c^	200.3 ± 11.0 ^b^	223.5 ± 3.2 ^a^	***	***
	Mal3g ^8^	2720.5 ± 90.5 ^d^	3759.5 ± 103.4 ^c^	4406.6 ± 60.8 ^a^	4245.9 ± 77.3 ^b^	4256.2 ± 104.6 ^d^	4655.1 ± 87.0 ^c^	6389.5 ± 254.8 ^a^	6106.0 ± 50.6 ^b^	***	***
Flavonols	M3Og ^9^	53.4 ± 2.3 ^d^	72.3 ± 3.6 ^c^	89.4 ± 3.8 ^b^	100.2 ± 3.5 ^a^	50.1 ± 5.5 ^c^	61.6 ± 0.9 ^b^	90.4 ± 3.0 ^a^	84.6 ± 2.7 ^a^	***	**
	Rut ^10^	50.0 ± 5.2 ^a^	38.7 ± 0.8 ^b^	31.5 ± 3.3 ^c^	23.6 ± 1.4 ^d^	14.4 ± 1.0 ^b^	14.7 ± 0.6 ^b^	16.6 ± 0.3 ^a^	14.8 ± 0.6 ^b^	***	***
	Hyper ^11^	23.5 ± 3.4 ^a^	20.5 ± 1.3 ^a^	21.3 ± 3.1 ^a^	22.2 ± 3.5 ^a^	12.1 ± 1.0 ^d^	20.7 ± 0.3 ^c^	28.3 ± 2.5 ^b^	46.2 ± 0.8 ^a^	***	***
	Q3Og ^12^	320.1 ± 7.9 ^a^	319.1 ± 1.9 ^a^	249.7 ± 4.8 ^b^	245.0 ± 11.7 ^b^	170.4 ± 7.9 ^d^	195.5 ± 5.3 ^c^	239.7 ± 0.6 ^b^	326.9 ± 3.6 ^a^	***	***
	K3Og ^13^	21.7 ± 1.8 ^a^	18.4 ± 0.8 ^b^	22.1 ± 0.2 ^a^	16.1 ± 2.2 ^b^	8.8 ± 1.8 ^d^	20.6 ± 1.5 ^c^	28.6 ± 2.6 ^b^	51.7 ± 1.0 ^a^	***	***
	Iso3g ^14^	24.8 ± 1.9 ^c^	25.9 ± 1.8 ^c^	30.8 ± 3.2 ^b^	36.0 ± 1.8 ^a^	15.3 ± 1.3 ^d^	23.5 ± 2.0 ^c^	37.2 ± 2.3 ^b^	48.7 ± 4.2 ^a^	ns	***
Flavan-3-ols monomeric forms	GC ^15^	3.5 ± 0.4 ^d^	5.0 ± 0.2 ^c^	6.7 ± 0.6 ^b^	8.8 ± 0.4 ^a^	3.2 ± 0.4 ^a^	2.3 ± 0.1 ^b^	3.2 ± 0.4 ^a^	1.2 ± 0.1 ^c^	***	***
	EGC ^16^	92.6 ± 3.7 ^a^	88.9 ± 2.1 ^a b^	84.2 ± 4.2 ^b^	60.0 ± 3.8 ^c^	107.8 ± 3.1 ^a^	93.4 ± 2.4 ^b^	103.7 ± 5.8 ^a^	83.0 ± 0.9 ^c^	***	**
	C ^17^	43.2 ± 1.8 ^a^	44.4 ± 4.0 ^a^	31.8 ± 2.8 ^b^	28.1 ± 2.3 ^b^	34.0 ± 1.6 ^a^	28.6 ± 2.0 ^b^	32.3 ± 1.5 ^a^	26.0 ± 0.9 ^b^	***	***
	EC ^18^	4.2 ± 0.3 ^c^	7.0 ± 0.7 ^b^	8.6 ± 0.5 ^a^	7.5 ± 1.7 ^ab^	4.3 ± 1.2 ^b^	4.2 ± 0.5 ^b^	15.4 ± 2.5 ^a^	17.6 ± 1.8 ^a^	***	***
Flavan-3-ols dimeric forms	B1	85.3 ± 2.3 ^a^	69.4 ± 5.3 ^b^	72.4 ± 0.8 ^b^	46.3 ± 3.3 ^c^	154.7 ±3.1 ^a^	136.7 ± 5.5 ^b^	147.1 ± 6.5 ^a^	118.5 ± 1.4 ^c^	***	ns
	B2	12.7 ± 0.5 ^b^	15.5 ± 2.1 ^a^	12.7 ± 0.8 ^b^	13.1 ± 1.7 ^ab^	10.8 ± 0.4 ^c^	11.3 ± 0.3 ^c^	11.9 ± 0.3 ^b^	12.9 ± 0.4 ^a^	***	**
	B3	15.0 ± 0.8 ^a^	15.4 ± 1.6 ^a^	11.6 ± 1.0 ^b^	10.4 ± 0.7 ^b^	14.5 ± 0.8 ^a^	11.6 ± 0.5 ^b^	14.3 ± 1.6 ^a^	10.2 ± 0.4 ^b^	ns	***
	B4	14.3 ± 0.6 ^b^	15.9 ± 0.9 ^a^	14.5 ± 0.9 ^ab^	14.3 ± 0.5 ^b^	17.4 ± 0.2 ^a^	16.3 ± 0.2 ^b^	17.6 ± 0.7 ^a^	15.4 ± 0.2 ^c^	***	**

Two-way ANOVA showing mean separation of the flavonoid components of Plavac Mali harvested at four different dates ^1^ HD (H1, H2, H3 and H4) at two locations (^2^ L; Split and Zadar) and the ^3^ I interactive effect of harvest date and location (ns: not significant; ** *p* ≤ 0.01; *** *p* ≤ 0.001). Mean values ± standard deviation (*n* = 3) within the same line, followed by different letters indicate significant differences according to Fisher’s LSD test at *p* ≤ 0.05 between different harvest dates, separately at each growing location as determined by one way ANOVA. ^4^ Del3g—delphinidin-3-*O*-glucoside, ^5^ Cya3g—cyanidin-3-*O*-glucoside, ^6^ Pet3g—Petunidin-3-*O*-glucoside, ^7^ Peo3g—Peonidin-3-*O*-glucoside, ^8^ Mal3g—malvidin-3-*O*-glucoside, ^9^ M3Og—myricetin-3-*O*-glucoside, ^10^ Rut—rutin, ^11^ Hyper—hyperoxide, ^12^ Q3Og—Quercetin-3-*O*-glucoside, ^13^ K3Og—Kaempferol-3-*O*-glucoside, ^14^ Iso3g—Isorhamnetin-3-*O*-glucoside,^15^ GC—gallocatechin, ^16^ EGC—epigallocatechin, ^17^ C—catechin, ^18^ EC—epicatechin. Concentrations of flavonoids are presented in units of mg kg^−1^ dry weight.

**Table 3:** Flavonoid composition (mg kg^−1^ dry weight) of Plavac Mali grape seed at four different harvest dates at two locations.

Split						Zadar					
HD ^1^		H1	H2	H3	H4	H1	H2	H3	H4	L ^2^	I ^3^
Flavan-3-ols monomeric forms	ECG ^4^	93 ± 21 ^a^	108 ± 2 ^a^	54 ± 17 ^b^	46 ± 17 ^b^	184 ± 4 ^a^	152 ± 35 ^a^	160 ± 25 ^a^	108 ± 12 ^b^	***	ns
	GC ^5^	160 ± 7 ^a^	166 ± 9 ^a^	152 ± 8 ^a^	137 ± 14 ^b^	166 ± 9 ^a^	168 ± 12 ^a^	167 ± 23 ^a^	164 ± 5 ^a^	*	ns
	EGC ^6^	205 ± 14 ^a^	215 ± 15 ^a^	210 ± 8 ^a^	178 ± 11 ^b^	282 ± 25 ^a^	261 ± 32 ^a^	259 ± 45 ^a^	232 ± 20 ^a^	***	ns
	C ^7^	7453 ± 393 ^a^	6811 ± 264 ^a^	4799 ± 277 ^b^	4507 ± 799 ^b^	19,142 ± 822 ^a^	17,901 ± 848 ^ab^	15,223 ± 2246 ^c^	15,410 ± 993 ^bc^	***	ns
	EC ^8^	6670 ± 157 ^a^	6821 ± 105 ^a^	5775 ± 40 ^b^	5292 ± 890 ^b^	14,871 ± 548 ^a^	13,664 ± 854 ^a^	12,888 ± 1685 ^a^	12,793 ± 1188 ^a^	***	ns
Flavan-3-ols dimeric forms	B1	3625 ± 219 ^ab^	3929 ± 294 ^a^	3520 ± 178 ^ab^	3270 ± 566 ^b^	5599 ± 485 ^a^	5486 ± 545 ^a^	5644 ± 1291 ^a^	5919 ± 397 ^a^	***	ns
	B2	2864 ± 196 ^a^	3201 ± 171 ^a^	3030 ± 77 ^a^	2763 ± 455 ^a^	4460 ± 442 ^a^	4230 ± 397 ^a^	4452 ± 934 ^a^	4518 ± 326 ^a^	***	ns
	B4	969 ± 61 ^a^	970 ± 48 ^a^	843 ± 32 ^ab^	753 ± 121 ^b^	1427 ± 96 ^a^	1331 ± 122 ^a^	1278 ± 269 ^a^	1310 ± 86 ^a^	***	ns

Two-way ANOVA showing mean separation of the flavonoid components of Plavac Mali harvested at four different dates ^1^ HD (H1, H2, H3 and H4) at two locations (^2^ L; Split and Zadar) and the ^3^ I interactive effect of harvest date and location (ns: not significant; * *p* ≤ 0.05; *** *p* ≤ 0.001). Mean values ± standard deviation (*n* = 3) within the same line followed by different letters indicate significant differences according to Fisher’s LSD test at *p* ≤ 0.05 between different harvest dates, separately at each growing location as determined by one-way ANOVA. ^4^ ECG-epicatechin-3-*O*-gallate, ^5^ GC-gallocatechin, ^6^ EGC-epigallocatechin, ^7^ C-catechin, ^8^ EC-epicatechin. Concentrations of flavonoids are presented in units of mg kg^−1^ dry weight.

**Table 4:** Grape variables of green berries separated from H1 harvest date at Split and Zadar vineyards.

Quality Variable		Split	Zadar	Significance
Morphological parameters	Total mass (150) berries (g)	104.11 ± 18.2 ^b^	152.78 ± 12.5 ^a^	* (0.0187)
	Mass 1 berry (g)	0.69 ± 0.12 ^b^	1.02 ± 0.08 ^a^	* (0.0187)
	Total number of seeds in 150 berries	195.33 ± 6.51 ^a^	172 ± 10.15 ^b^	* (0.0285)
	Total seed mass (g)	4.16 ± 0.58 ^a^	2.87 ± 0.29 ^b^	* (0.0270)
	Average mass of 100 seeds (g)	2.33 ± 0.45	1.66 ± 0.28	ns
Primary metabolites	TSS ^1^(°Brix)	10.71 ± 0.26 ^a^	8.94 ± 0.15 ^b^	*** (0.0004)
	pH	2.87 ± 0.02	2.87 ± 0.02	ns
	Potassium (g L^−1^)	747.96 ± 152.4	591.2 ± 23.2	ns
	TA ^2^	18.06 ± 0.65	18.51 ± 0.34	ns
	Citric acid (g L^−1^)	0.54 ± 0.06 ^a^	0.39 ± 0.01 ^b^	* (0.0133)
	Tartaric acid (g L^−1^)	10.42 ± 0.33	10.63 ± 0.24	ns
	Malic acid (g L^−1^)	7.88 ± 0.34 ^b^	10.67 ± 0.21 ^a^	*** (0.0003)
	Succinic acid (g L^−1^)	1.68 ± 0.04	nd	nd
	Glucose (g L^−1^)	49.25 ± 31.74	47.39 ± 19.81	ns
	Fructose (g L^−1^)	41.2 ± 24.52	38.81 ± 11.55	ns
	Panopa (g L^−1^)	62.27 ± 24.92 ^b^	161.02 ± 54.08 ^a^	* (0.0453)
**Flavonols**	Rutin	76.82 ± 4.81 ^a^	27.88 ± 0.61 ^b^	**** (0.0001)
	Hyper	25.18 ± 0.91	nd	nd
	Q3Og ^3^	591.31 ± 16.64 ^a^	422.05 ± 19.69 ^b^	*** (0.0003)
	K3Og ^4^	18.25 ± 2.73	nd	nd
**Flavan-3-ol monomers (skin)**	GC ^5^	12.21 ± 0.38	13.81 ± 1.13	ns
	EGC ^6^	252.64 ± 7.24	236.69 ± 9.82	ns
	C ^7^	126.11 ± 5.31 ^a^	82.22 ± 3.23 ^b^	*** (0.0003)
	EC ^8^	7.25 ± 0.8	nd	nd
**Dimers**	B1	58.02 ± 2.56	52.35 ± 2.88	ns
	B2	12.61 ± 0.51	12.07 ± 1.96	ns
	B4	11.10 ± 1.28	10.99 ± 1.39	ns
**Flavan-3-ol monomers (seed)**	ECG ^9^	27.89 ± 9.82 ^b^	110.36 ± 1.44 ^a^	**** (0.0001)
	GC	125.04 ± 17.10	96.08 ± 6.34	ns
	EGC	103.11 ± 20.55 ^b^	185.90 ± 7.14 ^a^	** (0.0027)
	C	7161.02 ± 528.24 ^b^	10,197.26 ± 997.97 ^a^	** (0.0096)
	EC	8720.9 ± 336.7 ^a^	6885.45 ± 318.68 ^b^	** (0.0024)
**Dimers**	B1	2686.73 ± 398.33	2028.01 ± 105.89	ns
	B2	3169.40 ± 673.1 ^a^	1780.64 ± 57.84 ^b^	* (0.0236)
	B4	804.19 ± 81.63	715.99 ± 23.54	ns

Mean values ± standard deviation (*n* = 3) within the same line followed by different letters indicate significant differences according to Fisher’s LSD test at *p* ≤ 0.05 between green berries, separately at each growing location as determined by one-way ANOVA (ns: not significant; * *p* ≤ 0.05; ** *p* ≤ 0.01; *** *p* ≤ 0.001). ^1^ TSS—total soluble solids, ^2^ TA—total acidity expressed as (g L^−1^) tartaric acid equivalents, ^3^ Q3Og—Quercetin-3-*O*-glucoside, ^4^ K3Ogv—Kaempferol-3-*O*-glucoside, ^5^ GC-gallocatechin, ^6^ EGC-epigallocatechin, ^7^ C-catechin, ^8^ EC-epicatechin, ^9^ ECG—epicatechin-3-*O*-gallate. Concentrations of flavonoids are presented in units of mg kg^−1^ dry weight.

**Table 5:** The change in values of CIELab color parameters and CIRG index of berry skin color at four different harvest dates per location.

	Split				Zadar				L	I
HD	H1	H2	H3	H4	H1	H2	H3	H4		
L* ^1^	25.15 ± 1.51 ^a^	25.17 ± 1.06 ^a^	25.00 ± 0.99 ^a^	24.37 ± 1.30 ^b^	25.07 ± 0.97 ^a^	24.70 ± 1.05 ^b^	24.29 ± 0.92 ^c^	23.61 ± 0.92 ^d^	***	***
a* ^2^	4.37 ± 2.68 ^a^	2.87 ± 1.82 ^b^	1.99 ± 1.12 ^c^	1.90 ± 1.06 ^c^	2.35 ± 1.04 ^a^	1.87 ± 1.04 ^b^	1.47 ± 0.55 ^c^	1.60 ± 0.64 ^c^	***	***
b* ^3^	0.73 ± 0.92 ^a^	0.35 ± 0.59 ^b^	0.21 ± 0.47 ^c^	0.35 ± 0.47 ^b^	0.61 ± 0.41 ^a^	0.46 ± 0.35 ^b^	0.45 ± 0.31 ^b^	0.42 ± 0.35 ^b^	**	***
C ^4^	4.46 ± 2.78 ^a^	2.94 ± 1.83 ^b^	2.06 ± 1.11 ^c^	1.99 ± 1.05 ^c^	2.45 ± 1.08 ^a^	1.95 ± 1.06 ^b^	1.57 ± 0.55 ^c^	1.69 ± 0.63 ^c^	***	***
h° ^5^	8.65 ± 7.11 ^a^	6.35 ± 12.67 ^b^	6.29 ± 15.32 ^b^	10.56 ± 16.53 ^a^	14.23 ± 7.71 ^b^	14.03 ± 9.78 ^b^	17.00 ± 11.71 ^a^	14.84 ± 13.78 ^b^	***	***
CIRG ^6^	5.88 ± 0.71 ^c^	6.22 ± 0.64 ^b^	6.43 ± 0.60 ^a^	6.45 ± 0.68 ^a^	6.04 ± 0.45 ^c^	6.25 ± 0.53 ^b^	6.32 ± 0.53 ^b^	6.54 ± 0.56 ^a^	ns	**

Two-way ANOVA showing mean separation of the CIELab color parameters Plavac Mali harvested at four different dates (H1, H2, H3 and H4) at two locations (L; Split and Zadar) and the interactive effect (I = HD × L) of these factors (ns: not significant; ** *p* ≤ 0.01; *** *p* ≤ 0.001). Mean values ± standard deviation (*n* = 3) within the same line followed by different letters indicate significant differences according to Fisher’s LSD test at *p ≤ 0.05* between different harvest dates, separately at each growing location as determined by one-way ANOVA. ^1^ L*—Lightness, ^2^ a*—red/green, ^3^ b*—yellow/blue, ^4^ C*—chroma, ^5^ h°—hue angle, ^6^ CIRG—color index for red grapes.

**Table 6:** Pearson correlation coefficients CIELab color variables (L*, a*, b*, C, h and CIRG) and concentration of individual anthocyanin-3-*O*-monoglucosides.

CIELab	Del3g ^1^	Cya3g ^2^	Pet3g ^3^	Peo3g ^4^	Mal3g ^5^
L* ^6^	−0.76 ***	−0.81 ***	−0.80 ***	−0.77 ***	−0.76 ***
a* ^7^	−0.90 ***	−0.53 **	−0.82 ***	−0.53 **	−0.83 ***
b* ^8^	−0.46 *	−0.33 ^n.s.^	−0.27 ^n.s.^	−0.23 ^n.s.^	−0.30 ^n.s.^
C ^9^	−0.89 ***	−0.53 **	−0.81 ***	−0.53 **	−0.82 ***
H ^10^	0.57 **	0.34 ^n.s.^	0.70 ***	0.46 *	0.67 ***
CIRG ^11^	0.73 ***	0.65 ***	0.61 **	0.55 **	0.61 **

^1^ Del3g—delphinidin-3-*O*-glucoside, ^2^ Cya3g—cyanidin-3-*O*-glucoside, ^3^ Pet3g—petunidin-3-*O*-glucoside, ^4^ Peo3g—peonidin-3-*O*-glucoside, ^5^ Mal3g—malvidin-3-*O*-glucoside, ^6^ L*—Lightness, ^7^ a*—red/green, ^8^ b*—yellow/blue, ^9^ C*—chroma, ^10^ h°—hue angle, ^11^ CIRG—color index for red grapes *—significant at *p* < 0.05; ** *p* < 0.01; *** *p* < 0.001; n.s.—not significant.

## Supplementary Materials

The following are available online at https://www.mdpi.com/2304-8158/9/9/1155/s1, Figure S1: Monthly average temperature and rainfall for the two studied locations, Split (Duilovo) and Zadar (Baštica), Figure S2: HPLC chromatogram of individual anthocyanin monoglucosides in Plavac Mali grape skin extract. The peaks correspond to 1 delphinidin-3-*O*-glucoside, 2 cyanidin-3-*O*-glucoside, 3 petunidin-3-*O*-glucoside, 4 malvidin-3-*O*-glucoside, 5 peonidin-3-*O*-glucoside.
