# Supplementary material for: Extended Harvest Date Alter Flavonoid Composition and Chromatic Characteristics of Plavac Mali (Vitis vinifera L.) Grape Berries
Source: Foods. 2020 Aug 21;9(9):1155. doi: 10.3390/foods9091155 (PMC7555124; doi:10.3390/foods9091155)
Supplement: Supplementary file 1 [file foods-09-01155-s001.pdf]

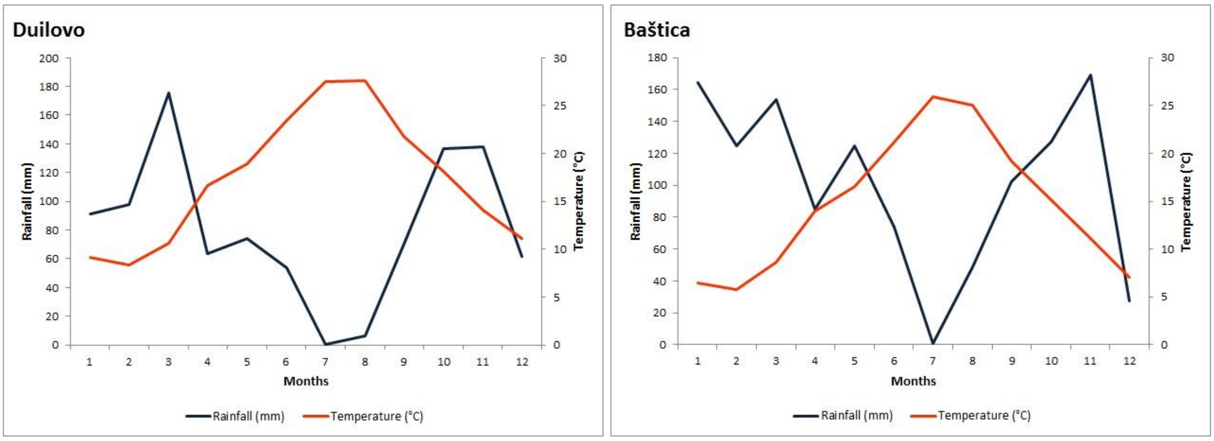

Figure S1. Monthly average temperature and rainfall for the two studied locations, Split (Duilovo) and Zadar (Baštica)

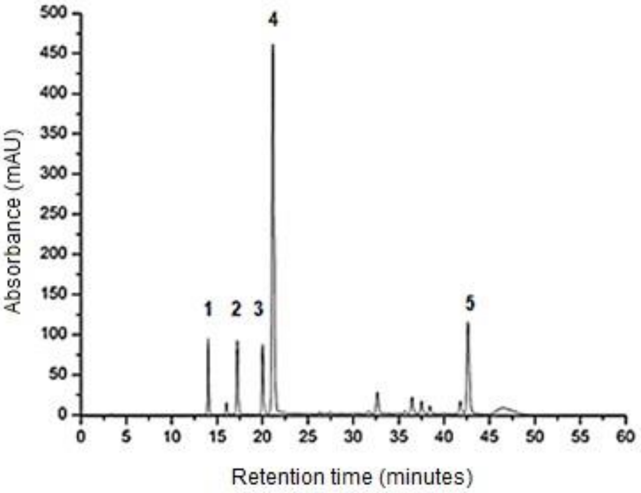

Figure S2. HPLC chromatogram of individual anthocyanin monoglucosides in Plavac Mali grape skin extract. The peaks correspond to: 1 delphinidin-3-*O*-glucoside, 2 cyanidin-3-*O*-glucoside, 3 petunidin-3-*O*-glucoside, 4 malvidin-3-*O*-glucoside, 5 peonidin-3-*O*-glucoside.
